# Effect of Korean Dental Hygienists’ Experiences Related to the Elderly on Their Perception of Human Rights Violations of the Elderly: A Cross-Sectional Study

**DOI:** 10.3390/ijerph19063376

**Published:** 2022-03-13

**Authors:** Kyeung-Ae Jang, Yu-Rin Kim

**Affiliations:** Department of Dental Hygiene, Silla University, Busan 46958, Korea; jka@silla.ac.kr

**Keywords:** aged, knowledge, oral health, health behavior, human rights

## Abstract

Background: Korean society has become an elderly society at an unprecedented rate, and the associated social and economic problems are very serious. Maintaining a healthy oral condition is important for older people’s well-being and quality of life, and is essential for healthy ageing. Therefore, the purpose of this study was to investigate the effect of dental hygienists’ experiences related to the elderly on their perception of human rights violations toward the elderly. Methods: This study was conducted through an IRB review at Silla University in Korea (No. 1041449-202012-HR-001). For about a month after 1 February 2021, the survey questionnaire was distributed to dental hygienists living in South Korea, and 153 people who completed and sent back the questionnaires were selected as the final subjects. The items on the questionnaire consisted of perception of human rights violations for the elderly and experiences related to the elderly. A polyserial correlation analysis was performed to confirm the relevance of each factor, and a multiple regression analysis was performed to identify the factors of elderly-related experiences that affect the perception of human rights violations against the elderly. Results: All three experiences related to the elderly (the experience of receiving education related to the elderly, the experience of volunteer work related to the elderly, and the experience of being interested in issues related to the elderly) were highly related to the perception of violations of financial human rights of the elderly (*p* < 0.01). In addition, the experience of education related to the elderly showed a high perception of psychological violations of the human rights of the elderly (R = 0.405, *p* < 0.01), and the experience of volunteer activities related to the elderly showed a high perception of neglectful violations of human rights of the elderly (R = 0.277, *p* < 0.01). Conclusions: In this study, it was confirmed that there is a relationship between dental hygienists’ experiences related to the elderly and their perception of human rights violations in the elderly. Therefore, dental hygienists should do their best to manage the elderly’s oral health by raising awareness of the human rights violations against the elderly through the elderly professional course based on their experience with the elderly.

## 1. Introduction

The United Nations (UN) defined a post-aged society or a super-aged society as that when the proportion of the population aged 65 and above accounts for more than 20% of the total population [1]. The elderly population is expected to be larger than the population of children under five years within a decade, and to double by 2040 to eventually reach as many as two billion individuals by 2050, by which time it will represent more than 20% of the world’s population [2]. The population belonging to WHO’s South-East Asia region is also ageing rapidly; for instance, the proportion of people aged 60 or above was 9.8% in 2017 and is expected to increase to 13.7% by 2030 and to 20.3% by 2050 [3]. Between 2019 and 2050, 9 out of the 10 countries with the largest percentage-point increase in the share of older persons in the world will be in East and South-East Asia. The largest increase is foreseen in the Republic of Korea (23%), followed by Singapore (20.9%) and Taiwan Province of China (19.9%) [1]. In the case of South Korea, among the elderly, the very old (above 80 years) are also the fastest-growing population, which is creating new challenges. A combination of increased life expectancy and reduced fertility is increasing the absolute number of old persons, even as it is boosting their relative share of the population. South Korea has already become an aged society, as the proportion of its population aged 65 and older exceeded 14% in 2018, and is projected to reach 20.3% by 2025, at which time it is expected to enter a super-aged society worldwide [4]. However, unlike countries that have long prepared for an elderly society, including Japan (with a 27.3% elderly population) and Switzerland (with a 23.4% elderly population), Korea has earned an elderly society status at an unprecedented rate [5,6]. Thus, the related social and economic problems that it is experiencing are very serious. This trend is not unique to Korea, however; the increase in the aged population in every corner of the world has led to social and health problems that include various forms of violence perpetrated against older adults.

The human rights of older persons is a topic that has been neglected for a long time. In at least some countries, it is a dimension that is being increasingly considered, but often haphazardly. The human rights of the elderly were first mentioned in 1948 in the UN’s Declaration of the Rights of the Elderly. The declaration focused on 10 social rights of the elderly: the right to receive assistance, to work, to food, to clothing, to shelter, to care for their physical and mental health, to leisure, to safety, to security, and to respect [7]. Additionally, in 1991, the UN enacted the United Nations Principles for Older Persons and presented 18 specific principles that governments should consider in each of the five areas of independence, participation, care, self-fulfillment, and dignity [8]. Internationally, however, the debate on the predicament of senior citizens in terms of rights is only beginning. For example, it was only in 2009 that the Human Rights Council Advisory Committee recommended a study on the “need to protect the human rights of the older person in the context of a human rights framework” [9]. Rim and Yun [10] emphasized the importance of the elderly’s perception of human rights violations against them and, notably, their caregivers’ perceptions of such violations. Rim and Yun further pointed out that the perception of human rights violations against the elderly is lacking and that human rights violations against the elderly have diverse and complex causes such as individual, family, and social situations. In Korea in 2016, there were 6,811 reports of elder abuse, the most serious form of human rights violation, of which 40.1% was psychological abuse, 31.3% was physical abuse, 11.4% was neglect by others, 7.7% was negligence of self, 7.2% was financial abuse, 1.3% was sexual abuse, and 1.0% was abandonment [11]. These figures suggest that there is an urgent need for institutional arrangements at the national and social levels to guarantee the protection of the human rights of the elderly. The research of Kim et al. [12] found that lack of awareness of human rights and abuse of the elderly are highly related. In another work, Kim et al. [13] found that the higher the awareness of human rights is, the lower the abuse is. As such, awareness of the human rights of the elderly is a very important part of the life of a healthy elderly person. 

The oldest elderly people are often described as a frail and multimorbid group [14]. For the individual, frailty increases vulnerability due to diminished strength, endurance, and physiological function [15]. Frail individuals have a higher risk of developing poor oral health due to limitations in their ability to perform self-care and difficulties in visiting dental health care clinics [16]. In the elderly, mastication discomfort and dysphagia due to tooth loss lead to nutritional deficiency, which is related to mental health and quality of life as well as physical activity [17]. 

Good oral health is important for older people’s well-being and quality of life, and is essential for healthy ageing [18]. However, there is insufficient knowledge about how older people view their oral health and oral care [19]. Koistinen et al. found that 85% of the older people from 36 Swedish STC units were satisfied with their oral health, even though clinical assessments by registered dental hygienists showed that 77% of these individuals had oral problems such as coating and food debris [20]. As such, the elderly lack accurate awareness of their oral health. Therefore, dental hygienists should understand the characteristics of the elderly and try to take care of their oral care. In Europe, the oral health of the elderly has improved, increasing the number of elderly people who maintain natural teeth for life [21]. In Sweden, approximately 60% of people aged 80–89 years have 20 or more teeth [22]. In line with this, Korean dental hygienists should also focus on oral care by identifying the characteristics of the rapidly increasing number of the elderly.

In order to become a suitable dental hygienist in this aging era, it is necessary to understand the characteristics of the elderly through various experiences with them and to know the importance of human rights of the elderly. Based on these experiences, dental hygienists should also strive to promote oral health that can improve the human rights and quality of life of the elderly. Therefore, the purpose of this study is to confirm the effect of dental hygienists’ experiences related to the elderly on the perception of human rights violations of the elderly.

## 2. Materials and Methods

### 2.1. Study Subjects and Method 

**Figure 1:**
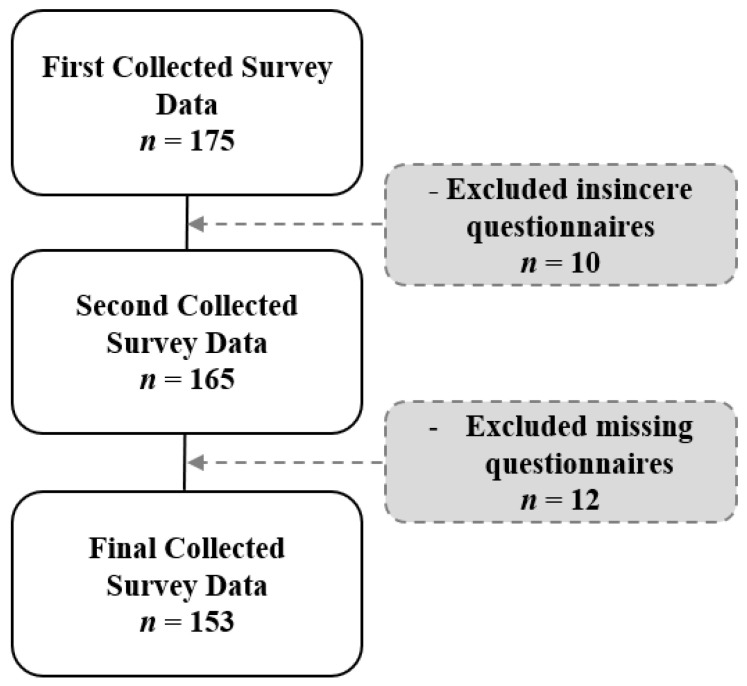
Flowchart of the study.

This study was conducted with the approval of the Bioethics Review Committee of Silla University (1041449-202012-HR-001). The subjects of this study were recruited using the convenience screening method for dental hygienists in Korea, and the survey period was conducted from 1 February to 29 May 2021. For ethical consideration of the subject of the study, a structured self-contained survey was conducted on each subject who signed the consent form. The survey method was self-filling, and the dental hygienists filled out the questionnaire themselves. Based on Cohen’s power analysis, G*power 3.1.3 [23] was used, and the minimum sample required under the condition of a 5% significance level (two-tailed), a power of 80%, and an effect size of 0.5 was used. We planned to retrieve a total of 151 questionnaires, but 175 questionnaires were distributed in consideration of the dropout rate. As 22 questionnaires were not answered or were insincerely answered, 153 duly accomplished questionnaires were finally used (Figure 1).

### 2.2. Study Tools 

#### 2.2.1. Questionnaire of Demographic 

Sex and age were investigated as demographic characteristics of study subjects, and the ages were divided into ‘under 25’, ‘25–29 years’, ‘30–34 years’, ‘35–39 years’, and ‘over 39 years’. The work experience of dental hygienists was classified into ‘under 1 year’, ‘1–2 years’, ‘3–4 years’, ‘5–6 years’, and ‘over 7 years’.

#### 2.2.2. Questionnaire on the Perception of Human Rights Violations against the Elderly

The measurement tool for the perception of human rights violations against the elderly that was used in this study was based on the United Nations Principles for Older Persons [8]. It was a modified version of the questionnaire that Kang and Lim [24] used in their study. The questionnaire had a total of 22 questions that were grouped according to six dimensions of human rights violations against the elderly: psychological, financial, physical, sexual, neglect of others, and neglect of self. A detailed description of each item is presented in Table 1. For each item, ‘not a human rights violation’ was rated as 1 point, ‘minor human rights violation’ as 2 points, ‘serious human rights violation’ as 3 points, and ‘very serious human rights violation’ as 4 points, and a high score means that the dental hygienist’s perception of human rights violations against the elderly is high. The Cronbach’s α was 0.804 for the perception of human rights violations against the elderly. 

#### 2.2.3. Questionnaire of Experiences Related to the Elderly

As for the measurement tool for elderly-related experiences, a modified version of the questionnaire that Jang and Heo [25] used in their study was used. The questions were grouped into three categories: experience of receiving education related to the elderly (Do you have any experience of receiving education related to the elderly at a university or an institution related to the elderly?), experience of volunteer work related to the elderly (Do you have any experience of volunteering for the elderly at an institution related to the welfare of the elderly?), and experience of being interested in issues related to the elderly (Have you ever been interested in the problems of the elderly?). Each question was answered with either “experienced” or “inexperienced”, and the higher the score was, the higher the experience with the elderly was. Education and volunteer activities related to the elderly refer to experiences at universities or elderly-related facilities, and interest in the elderly includes an interest in loss of role and leisure problems of the elderly, economic poverty problems, diseases and health protection problems, loneliness and alienation problems, etc.

Exploratory factor analysis was used to verify the validity of the questionnaire, and Bartlett’s identity matrix check and KMO values were analyzed by selecting items with a sample fit of 0.5 or higher. The Cronbach’s α was 0.653 for the elderly-related experience. The reliability coefficients were all higher than 0.6, which indicates the high internal consistency of the questionnaire tool.

### 2.3. Statistical Analysis

For the data analysis, the IBM SPSS Statistics (ver. 26.0 from SPSS Inc., Chicago, IL, USA) statistical program was used. The general characteristics of the study subjects were tabulated according to frequency and percentage, and the perception of human rights violations against the elderly according to experiences related to the elderly was analyzed through an independent t-test. A polyserial correlation analysis [26] was performed to confirm the relevance of each factor, and a multiple regression analysis was conducted to identify the factors of elderly-related experiences that affect the perception of human rights violations against the elderly. As a result of confirming normality for the perception of human rights violations in the elderly in this study, skewness was −0.099 ± 0.196 and kurtosis was −0.301 ± 0.390; therefore, normality was satisfied.

## 3. Results

### 3.1. Demographic of the Dental Hygienists

Table 2 shows that among the dental hygienists, there were more female than male and the biggest age group was the 25–29 years group. The average age of dental hygienists was 28.24 years. Most of the dental hygienists had more than 7 years of work experience, followed by those with 3–4 years of work experience. 

### 3.2. Perceived Degrees of Violation of Human Rights of the Elderly According to the Experience of Receiving Education Related to the Elderly

Table 3 shows that the degree perception of human rights violations against the elderly was significantly higher if the respondent had received education related to the elderly (*p* < 0.001). Among the six sub-items of perception of human rights violations against the elderly, the greatest difference between the dental hygienists who were educated on the elderly and those who were not was found in ‘Financial’. The next order was ‘Psychological’, ‘Physical’, ‘Neglect of others’, ‘Neglect of self’, and ‘Sexual’ (*p* < 0.001).

### 3.3. Perceived Degrees of Violation of Human Rights of the Elderly According to the Experience of Volunteer Activities Related to the Elderly

Table 4 shows that the degree of awareness of human rights violations against the elderly was significantly higher if the respondent had experienced volunteer activities related to the elderly (*p* < 0.01). Among the six sub-items of perception of human rights violations against the elderly, the greatest difference between the dental hygienists who had experience volunteering with the elderly and those who did not was found in ‘Neglect of self’. The next order was ‘Financial’, ‘Sexual’, ‘Physical’, ‘Psychological’, and ‘Neglect of others’ (*p* < 0.001).

### 3.4. Perceived Degrees of Violation of Human Rights of the Elderly According to the Experience of Taking Interest in Issues related to the Elderly

Table 5 shows that the degree of perception of human rights violations against the elderly was significantly higher if the respondent had experience with taking interest in issues related to the elderly (*p* < 0.001). Among the six sub-items of perception of human rights violations against the elderly, the greatest difference between the dental hygienists who had experience taking an interest in issues related to the elderly and those who did not was found in ‘Psychological’. The next order was ‘Financial’, ‘Neglect of others’, ‘Physical’, ‘Neglect of self’, and ‘Sexual’ (*p* < 0.001). 

### 3.5. Correlation between Experience Related to the Elderly and Subfactors of the Perception of Human Rights Violations against the Elderly

Table 6 shows the correlation between experiences related to the elderly and the subfactors of the perception of human rights violations against the elderly. The greater the experience of education related to the elderly was, the higher the perception of human rights violations against the elderly was, except for the perception of ‘Sexual’ human rights violations (*p* < 0.05). The greater the experience of volunteering with the elderly was, the higher the perception of human rights violations against the elderly was, except for the perception of ‘Neglect of others’ human rights violations (*p* < 0.05). The greater the experience of interest in issues related to the elderly was, the higher the perception of all items regarding the human rights violations against the elderly was (*p* < 0.01). There was also a relationship among the subfactors of the perception of human rights violations against the elderly (*p* < 0.05).

### 3.6. Factors in Which Experiences Related to the Elderly Affect the Perception of Human Rights Violations against the Elderly 

Table 7 shows the effects of elderly-related experiences on perception of human rights violations against them. The multicollinearity analysis to confirm the suitability of the independent variables showed that the Variance Inflation Factor (VIF) coefficients of all the variables were 10 or less. The explanatory power of the regression model was 25.4%, and it was found to have a statistically significant model fit (*p* < 0.001). As a result of analyzing the influence of each independent variable by adjusting for age and work experience, the experience of receiving education related to the elderly (*p* < 0.001), the experience of taking an interest in issues related to the elderly (*p* < 0.01), and the experience of volunteering with the elderly (*p* < 0.05) all affected the perception of human rights violations against the elderly. The standardization coefficients were compared to confirm the influence of experiences related to the elderly on the perception of human rights violations against the elderly. The results showed that the experience of education related to the elderly (β = 0.274), the experience of taking an interest in issues related to the elderly (β = 0.272), and the experience of volunteer work related to the elderly (β = 0.186) influenced the perception of human rights violations against the elderly, in that order.

## 4. Discussion

Oral conditions in the elderly cause them to have a weak ability to chew food. This can lead to dysphagia, which reduces the quantity and quality of meals, and ultimately leads to poor overall health through nutritional deficiencies and weight loss [27,28]. In fact, in a research panel survey of 6935 aging people, it was found that in the case of the 65-year-old and older group, when their Geriatric Oral Health Assessment Index (GOHAI) [29] increased by 1 unit, their health satisfaction and quality of life significantly improved [30]. As such, oral and systemic health are closely related [31]. However, with increasing age, declining health, and dependence on care, older people are more likely to develop poor oral health [16]. Moreover, the number of older people in need of care is expected to increase as the population continues to age [32]. Therefore, as the role of oral health care for the elderly is more emphasized, it is important for dental hygienists to properly perceive the elderly [33]. Unlike nurses, dental hygienists rarely have access to knowledge about the elderly through university education courses in Korea. Meanwhile, as health insurance is applied to the elderly over 65 with implants and dentures, the number of elderly patients visiting the dentist is increasing [34]. In line with this trend, dental hygienists should raise awareness of the human rights of the elderly for better oral care of the elderly and increase their understanding of the elderly by undertaking more experiences with the elderly. Therefore, the purpose of this study is to make oral care more efficient by analyzing the effect of dental hygienists’ various experiences with the elderly on the perception of human rights violations of the elderly and understanding the characteristics of the elderly.

As a result of this study, among the three experiences related to the elderly, the highest perception of human rights violations among the elderly was associated with receiving education on the elderly. The next experience was interest in the problems of the elderly, followed by volunteering for the elderly. Therefore, in order to improve understanding of human rights violations for the elderly, it will be important to precede education on the characteristics of the elderly. These results were similar to those of Sim and Kim [35]. In addition, Lee et al. [36] reported that those who had received oral health guidance education for the elderly had better attitudes toward the elderly, as well as better psychological characteristics, family relationship characteristics, and judgmental thinking skills compared to those without such experience.

Violation of the human rights of the elderly means that the elderly do not take care of themselves or their caregivers neglect their duty to care for them, e.g., by inflicting physical, psychological, sexual, financial, or neglectful harm on the elderly [6]. The biggest difference between those dental hygienists who had received education on the elderly and those who had not received such education was in their perception of financial human rights violations. Therefore, dental hygienists will know that they have the right to protect the assets of the elderly through education related to the elderly. Additionally, the biggest difference between those dental hygienists who had experience in taking an interest in issues related to the elderly and those who had not had such experience was in their perception of psychological human rights violations. The biggest difference between those dental hygienists who had experienced volunteer work related to the elderly and those who had not had such experience was in their perception of “neglect” human rights violations. These results are consistent with the results of Kang and Im [24], in which the elderly volunteer experience had a high average score in the perception of psychological human rights violations. Such psychological and neglect-oriented human rights violations are difficult to recognize as abuse and to assess for damages, so dental hygienists should pay attention to elderly-related volunteer activities and problems related to the elderly. In the results of this study, all three experiences related to the elderly were highly related to the perception of violations of financial human rights violations against the elderly. In addition, the dental hygienists who had experienced education related to the elderly showed a high perception of psychological human rights violations against the elderly, and those who had experienced volunteer activities related to the elderly showed a high perception of neglect-oriented human rights violations against the elderly. These results are similar to the results of a study in the UK, where neglect (1.1%) was reported as the most common type of abuse, followed by financial (0.7%) and psychological abusee (0.4%) [37]. To properly recognize financial, psychological, and neglect-oriented abuses, which account for a high proportion of human rights violations against the elderly, dental hygienists should make efforts to have the three mentioned categories of experiences related to the elderly.

In Korea, oral health services are provided as long-term care insurance for the elderly, but there is no system for oral hygiene services paid at home. In fact, there are only five dental hygienists in the country who provide visiting nursing oral hygiene services [38]. Therefore, due to the increasing number of the elderly, an experts’ course on oral care for the elderly is required for dental hygienists working in dental clinics. Therefore, additional research on the elderly oral care expert course including experience related to the elderly is needed. Furthermore, if a curriculum related to geriatric dentistry is opened and ran in the university curriculum, it will be possible for dental hygienists to better understand the human rights of the elderly and the oral health status of the elderly when they are still students. The limitation of this study is that since the experiences related to the elderly were confirmed only through questionnaires for dental hygienists, care should be taken in generalizing them. An experimental study on the perception of human rights violations against the elderly by applying the experiences of dental hygienists related to the elderly is necessary in the future. In addition, further studies are needed to identify various factors that affect the elderly-related experiences of dental hygienists and their perception of human rights violations against the elderly. It is expected that the results of this study can be used as basic data for the development of programs that will provide dental hygienists elderly-related experiences as a means of enhancing their perception of human rights violations against the elderly.

## 5. Conclusions

The results of this study showed that the elderly-related educational experience of dental hygienists, their interest in problems related to the elderly, and their volunteer activities related to the elderly affected their perception of human rights violations against the elderly. Therefore, in order for dental hygienists to raise awareness of human rights violations of the elderly, they should receive education on specialized courses for the elderly, including experiences related to the elderly. These specialized courses for the elderly should be systematically offered to enrolled students in the Department of Dental Hygiene at each university. In addition, dental hygienists who have graduated from university, they should have to receive continuous education through the elderly professional course program from the Dental Hygienists Association.

## Figures and Tables

**Table 1:** Contents of 6 sub-factors for the perception of human rights violations in the elderly.

Item	Contents
Psychological	Do you think it is a violation of the human rights of the elderly when a family avoids living with the elderly or neglects to support them?
	Do you think it is a violation of the human rights of the elderly when family members do not visit the elderly living separately or are stingy in subsidizing living expenses?
	Do you think it is a violation of the human rights of the elderly when family members or guardians curse or get angry at the elderly?
	Do you think that it is a violation of human rights for the elderly when family members or guardians prevent the elderly from meeting with family, relatives and neighbors or participating in social activities?
Financial	Do you think that not giving allowances to the elderly is a violation of the human rights of the elderly?
	Do you think it is a violation of the human rights of the elderly when others use or take away the old people’s money or property without permission?
	Do you think it is a violation of the human rights of the elderly to falsely fill out bank loan documents, wills, contracts, etc. in the name of the elderly, or to force the elderly to sign?
Physical	Do you think that pinching, biting, or pulling the hair of the elderly is a violation of the human rights of the elderly?
	Do you think hitting the elderly with your feet or fists is a violation of the human rights of the elderly?
	Do you think it is a violation of the human rights of the elderly when someone presses the neck of the elderly?
	Do you think it is a violation of the human rights of the elderly to bind their limbs or body so that they cannot move?
	Do you think that having the elderly do work that they cannot or do not want is a violation of the human rights of the elderly?
Sexual	Do you think that it is a violation of the human rights of the elderly when an elderly person interferes with the relationship of the opposite sex with another elderly person?
	When an elderly person wants to remarry (twilight), do you think it is a violation of the human rights of the elderly when their grandchildren interfere?
	Do you think that being sexually assaulted by the elderly, such as sexual harassment and harassment, is a violation of the human rights of the elderly?
Neglect of others	Do you think it is a violation of the human rights of the elderly when family members or guardians do not do what they want or force them to do what they do not want?
	Do you think that it is a violation of the human rights of the elderly when the elderly alone have difficulties in daily life, such as eating and doing housework, but their family or guardians do not help?
	Do you think it is a violation of the human rights of the elderly when family members or guardians do not help the elderly even though it is difficult to make a living?
	Do you think it is a violation of the human rights of the elderly when the elderly do not feel well because they skip meals or do not take medicine?
Neglect of self	If the elderly cannot contact any of their family members or guardians, Do you think it is also a violation of human rights for the elderly to be indifferent to the elderly when they are ill?
	Do you think it is a violation of the human rights of the elderly if the elderly do not respond quickly to taking medicine or hospital treatment when they are sick?
	Do you think the suicide of the elderly is a violation of human rights?

**Table 2:** Demographic of the dental hygienists. Unit: *n (*%).

Variable		*n*	%
Sex	Male	5	3.3
	Female	148	96.7
Age	<25	33	21.6
	25–29	71	46.4
	30–34	32	20.9
	35–39	13	8.5
	>39	4	2.6
Age (M ± SE)		28.24 ± 4.80	
Work experience	<1 year	29	19.0
	1–2 years	27	17.6
	3–4 years	31	20.3
	5–7 years	17	11.1
	>7 years	49	32.0
Total		153	100.0

**Table 3:** Perceived degrees of violation of human rights of the elderly according to the experience of receiving education related to the elderly M ± SD.

Subfactors	Experience in Education Related to the Elderly		t	*p*
	Experienced (*n* = 39)	Inexperienced (*n* = 114)		
Psychological	3.22 ± 0.62	2.60 ± 0.61	5.436	<0.001 ***
Physical	3.93 ± 0.21	3.58 ± 0.66	5.034	0.001 **
Financial	3.50 ± 0.34	2.94 ± 0.57	7.381	<0.001 ***
Sexual	3.56 ± 0.51	3.13 ± 1.43	1.845	0.005 **
Neglect of others	3.03 ± 0.50	2.58 ± 0.79	4.120	<0.001 ***
Neglect of self	3.28 ± 0.81	2.73 ± 0.76	3.894	0.001 **
Total	3.44 ± 0.35	2.96 ± 0.56	6.444	<0.001 ***

By independent *t*-test, ** *p* < 0.01, *** *p* < 0.001.

**Table 4:** Perceived degrees of violation of human rights of the elderly according to the experience of volunteer activities related to the elderly M ± SD.

Subfactors	Experience of Volunteer Work Related to the Elderly		t	*p*
	Experienced (*n* = 98)	Inexperienced (*n* = 55)		
Psychological	2.84 ± 0.61	2.61 ± 0.74	2.015	0.047 *
Physical	3.76 ± 0.48	3.50 ± 0.75	2.334	0.022 *
Financial	3.20 ± 0.50	2.88 ± 0.64	3.211	0.002 **
Sexual	3.45 ± 1.45	2.88 ± 0.74	2.696	0.008 **
Neglect of others	2.78 ± 0.66	2.55 ± 0.88	1.748	0.084
Neglect of self	3.03 ± 0.79	2.57 ± 0.74	3.547	0.001 **
Total	3.20 ± 0.50	2.87 ± 0.59	3.521	0.001 **

By independent *t*-test, * *p* < 0.05, ** *p* < 0.01

**Table 5:** Perceived degrees of violation of human rights of the elderly according to the experience of taking interest in issues related to the elderly M ± SD.

Subfactors	Experience in Taking Interest in Issues Related to the Elderly		t	*p*
	Experienced (*n* = 77)	Inexperienced (*n* = 76)		
Psychological	3.02 ± 0.64	2.50 ± 0.60	5.177	<0.001 ***
Physical	3.83 ± 0.42	3.51 ± 0.71	3.440	0.001 **
Financial	3.29 ± 0.49	2.88 ± 0.58	4.632	<0.001 ***
Sexual	3.52 ± 1.60	2.96 ± 0.72	2.819	0.005 **
Neglect of others	2.95 ± 0.58	2.44 ± 0.82	4.366	<0.001 ***
Neglect of self	3.08 ± 0.78	2.65 ± 0.77	3.440	0.001 **
Total	3.30 ± 0.48	2.85 ± 0.53	5.470	<0.001 ***

By independent *t*-test, ** *p* < 0.01, *** *p* < 0.001.

**Table 6:** Correlation between experience related to the elderly and subfactors of the perception of human rights violations against the elderly.

Variables	1	2	3	4	5	6	7	8	9
1	1.000								
2	0.126	1.000							
3	0.311 **	0.264 **	1.000						
4	0.405 **	0.171 *	0.388 **	1.000					
5	0.258 **	0.209 **	0.267 **	0.526 **	1.000				
6	0.429 **	0.270 **	0.353 **	0.723 **	0.716 **	1.000			
7	0.149	0.214 **	0.224 **	0.256 **	0.322 **	0.319 **	1.000		
8	0.260 **	0.152	0.335 **	0.718 **	0.432 **	0.594 **	0.240 **	1.000	
9	0.302 **	0.277 **	0.270 **	0.557 **	0.453 **	0.509 **	0.161 *	0.610 **	1.000

By polyserial correlation coefficient, * *p* < 0.05, ** *p* < 0.01, 1: experience of education related to the elderly, 2: experience of volunteer work related to the elderly, 3: experience of taking an interest in issues related to the elderly, 4: perception of psychological human rights violations against the elderly, 5: perception of physical human rights violations against the elderly, 6: perception of financial human rights violations against the elderly, 7: perception of sexual human rights violations against the elderly, 8: perception of “neglect of others” human rights violations against the elderly, 9: perception of “neglect of self” human rights violations against the elderly.

**Table 7:** Factors that influence the effect of experiences related to the elderly on the perception of human rights violations against the elderly.

Independent Variables	B	S.E.	β	t	*p*	VIF
Constant	4.426	0.191		23.153	<0.001 ***	
Age	0.060	0.071	0.106	0.849	0.397	3.118
Work experience	0.024	0.046	0.066	0.531	0.596	3.137
Experience of education related to the elderly	0.349	0.095	0.274	3.669	<0.001 ***	1.130
Experience of volunteer work related to the elderly	0.215	0.085	0.186	2.533	0.012 *	1.090
Experience of taking an interest in issues related to the elderly	0.301	0.085	0.272	3.554	0.001 **	1.184

By the multiple regression analysis, * *p* < 0.05, ** *p* < 0.01, *** *p* < 0.001; Durbin-Watson’s = 1.732, F = 11.005, Adjusted R^2^ = 0.272. Dependence variables: age, work experience, experiences related to the elderly.

## Data Availability

The data presented in this study are available on request from the corresponding author.
